# Mindfulness-Based Stress Reduction for the Treatment of Vestibular Migraine: A Prospective Pilot Study

**DOI:** 10.7759/cureus.79225

**Published:** 2025-02-18

**Authors:** Eric J Formeister, James Mitchell, Roseanne Krauter, Ricky Chae, Adam Gardi, Maxwell Hum, Jeffrey D Sharon

**Affiliations:** 1 Head and Neck Surgery and Communication Sciences, Duke University School of Medicine, Durham, USA; 2 Osher Center for Integrative Medicine, University of California, San Francisco, USA; 3 Otolaryngology, Head, and Neck Surgery, University of California, San Francisco, USA; 4 Otolaryngology, Head, and Neck Surgery, University of Massachusetts Chan Medical School, Worcester, USA; 5 Otolaryngology, Head, and Neck Surgery, Drexel University College of Medicine, Philadelphia, USA; 6 Otolaryngology, Head, and Neck Surgery, University of Texas Health Science Center at Houston McGovern Medical School, Houston, USA

**Keywords:** dizziness, meditation, mindfulness, stress reduction, vertigo, vestibular migraine

## Abstract

Purpose

The purpose of this study was to describe the implementation and efficacy of a mindfulness-based stress reduction (MBSR) program for treating dizziness symptoms in subjects with vestibular migraine (VM).

Materials and methods

A non-controlled prospective cohort study of 20 adult English-speaking patients with a diagnosis of VM was undertaken at a single tertiary referral center. A virtual platform was used to administer an eight-week-long MBSR course with required reading materials and weekly 2.5-hour guided meditation and instructional sessions. Pre- and post-MBSR scores on the Dizziness Handicap Index (DHI), Cognitive Failures Questionnaire (CFQ), Patient Health Questionnaire-9 (PHQ-9), General Anxiety Disorder-7 (GAD-7), VM Patient Assessment Tool and Handicap Inventory (VM-PATHI), Patient Reported Outcome Measure Information System (PROMIS) Global Physical and Mental Health Forms, and daily vertigo severity scores were collected.

Results

Twenty participants (100.0% female; 70.0% (n=14) White; with an average age of 46.7 years (SD 16.3), completed the study. The DHI, CFQ, PHQ-9, GAD-7, VM-PATHI, and PROMIS scores all improved significantly after MBSR treatment compared to prior to treatment (all questionnaires, p<0.01 except for CFQ; p=0.03). Mean daily vertigo scores did not change significantly over time in the 24-day lead-in period (adjusted r^2 ^= 0.03; p=0.54) but decreased significantly over the eight-week MBSR treatment period (adjusted r^2^ = 0.32; p<0.001).

Conclusions

In this non-controlled, prospective pilot study, a MBSR program was highly effective for decreasing dizziness burden and improving measures of quality of life in subjects with VM. Future randomized controlled trials are warranted and forthcoming.

## Introduction

Vestibular migraine (VM) is one of the most common causes of dizziness worldwide and affects an estimated 2.7% of the US adult population [1]. Furthermore, approximately 30 to 50% of patients with migraines report a history of dizziness or vertigo associated with their migraines [2-4]. Survey studies indicate that quality of life is substantially impacted for those suffering from VM, with greater than half reporting work or school absenteeism over a one-year period due to their dizziness symptoms [1]. Current treatments for VM, such as tricyclic antidepressants, selective serotonin reuptake inhibitors, selective serotonin and norepinephrine reuptake inhibitors, beta-adrenergic blockers, calcium channel blockers, triptans, and antiepileptic, are largely chosen based on analogy for the treatment of migraine. However, the verification of treatment efficacy for the treatment of vestibular migraine has been limited by a lack of high-quality studies [5]. In fact, a 2015 Cochrane review found that there were no randomized controlled trials on which to base initial treatment choice for vestibular migraine [6]. Compounding this treatment dilemma, all of the currently available anti-migraine medications used in the prevention or treatment of vestibular migraine have significant side effects, which can limit up-titration to therapeutic doses and stable, long-term use. One such example is found in the administration of antiepileptics, in which a 2015 Cochrane review found that up to 20% of patients on the median dose of topiramate discontinued use due to intolerance of side effects [7]. Additionally, a prospective trial of vestibular migraineurs found a rate of medication non-adherence of 14-36% for multiple prophylactic agents [8]. Taken together, these findings highlight the need for further investigation into non-pharmacologic options that would potentially be better tolerated and more closely adhered to.

Based on ancient techniques, the concept of mindfulness and self-reflection for treating disease was originally classified as an "alternative" medicine. However, the last 20 years have seen a surge of scientific publications showing that mindfulness-based stress reduction (MBSR) has a foundation in neuroscience and cortical plasticity related to changes in several brain regions associated with emotional regulation, self-perspective, and response to threats [9]. The existing literature studying MBSR supports its efficacy in multiple chronic disease conditions associated with unpleasant sensations, including chronic anxiety, depression, and stress [10]. Additionally, chronic pain acceptance improved in a randomized study of patients with chronic back pain who received MSBR versus no intervention [11].

More promisingly, MBSR has shown benefits in treating migraine and dizziness. MBSR combined with pharmacotherapy has been shown to improve pain and quality of life (as measured with the 36-Item Short Form Survey) in the treatment of migraines and chronic tension-type headaches compared to pharmacotherapy alone [12]. Naber et al. evaluated multimodal treatment for dizziness from a variety of causes and reported improved physical and mental health, better functionality, less impairment, and fewer limitations from dizziness in those who underwent treatment that included mindfulness techniques [13].

Finally, vestibular migraines are associated with multiple comorbid conditions that are also amenable to treatment through MBSR techniques. For example, numerous studies support the association between VM and comorbid psychiatric illnesses such as depression and anxiety [1,14]. Pain catastrophizing, or a way of thinking that involves rumination, magnification, and feelings of helplessness related to a perceived or actual pain experience, may also degrade the quality of life and functioning. For example, the presence of catastrophization in migraine subjects was an independent predictor of functional impairment and impairment of quality of life after controlling for migraine characteristics and psychiatric comorbidities, suggesting that catastrophizing could be thought of as a separate entity from psychiatric diseases such as depression or anxiety [15]. The self-defeating nature of catastrophizing is the type of maladaptive behavior that MBSR courses seek to rectify. However, no studies exist that specifically address meditation therapies to help decrease dizziness symptoms in those with VM.

Given strong evidence of its utility for treating migraine in general, other dizziness disorders, and chronic conditions for which a maladaptive cycle of catastrophizing is a hallmark feature, this prospective pilot study sought to implement a guided, eight-week mindfulness-based meditation course to investigate its efficacy in improving quality of life and reducing dizziness symptoms in adult patients with VM. We hypothesized that, by disrupting the cycle of catastrophization that might be contributing to symptom persistence in vestibular migraine, patients might achieve decreased dizziness levels and improved overall quality of life.

This study was presented as an American Neurotology Society oral presentation at the annual Combined Otolaryngology Spring Meetings in Boston, MA, USA, May 2023.

## Materials and methods

Patients

This study was approved by the Institutional Review Board at the University of California, San Francisco (IRB No. 18-25365). Informed, written consent was obtained for each subject in accordance with the study institution. Consecutive adult patients greater than 18 years of age presenting to the study institution's otology and neurotology clinic for a new complaint of dizziness were evaluated. Those who met the Bárány Society's classification for probable or definite vestibular migraine [16] were asked to participate in an eight-week mindfulness course for the treatment of vestibular migraine. Inclusion criteria included fluency in the English language, access to high-speed internet, a personal computer and cellular phone, and willingness and ability to participate in eight weekly 2.5-hour sessions. Subjects with other overlapping peripheral vestibular diagnoses, such as active benign paroxysmal positional vertigo (BPPV) or Ménière's disease (MD), and a history of prior MBSR treatment were excluded. All patients were offered conservative treatment in addition to participation in the MBSR course, which included a comprehensive handout on migraine dietary trigger elimination diet and counseling on stress avoidance, exercising, and good sleep hygiene. Current use of any migraine prophylactic or abortive medication did not exclude participation. 

Mindfulness-based stress reduction (MBSR) course

**Figure 1:**
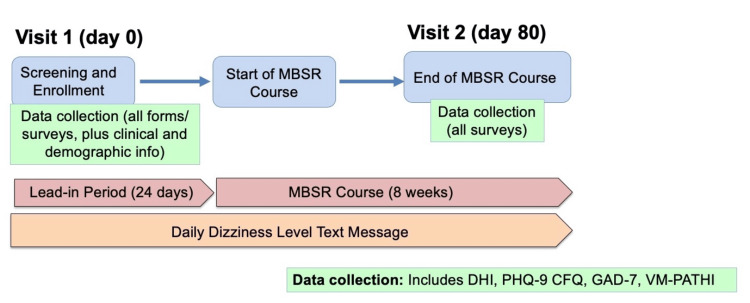
Flow chart outlining the prospective study design MBSR - mindfulness-based stress reduction; DHI - Dizziness Handicap Index; CFQ - Cognitive Failures Questionnaire; PHQ-9 - Patient Health Questionnaire-9; GAD-7 - General Anxiety Disorder-7, VM-PATHI - Vestibular Migraine Patient Assessment Tool and Handicap Inventory

Patients who met inclusion criteria were enrolled in an eight-week MBSR course at the University of California - San Francisco's Osher Center for Integrative Medicine (Figure 1). The course consisted of eight weekly sessions with a certified instructor trained in MBSR techniques (JM) over a virtual Zoom platform (Zoom Communications, San Jose, California). Additionally, participants read the book entitled Full Catastrophe Living by Jon Kabat-Zinn [17], who is credited as the creator of modern MBSR techniques. Sessions included guided yoga, breathing exercises, self-reflection, and meditation. The course fee of $435, as well as the cost of the required reading, was waived.

Main outcome measures

At enrollment, and prior to beginning the MBSR course, subjects completed dizziness-specific and quality-of-life surveys, including the Dizziness Handicap Index (DHI), Cognitive Failures Questionnaire (CFQ), Patient Health Questionnaire-9 (PHQ-9), General Anxiety Disorder-7 (GAD-7) questionnaire, the Vestibular Migraine Patient Assessment Tool and Handicap Inventory (VM-PATHI) [18], and the Patient Reported Outcomes Measurement Information System (PROMIS) mental and physical wellbeing surveys. In addition, daily text messages were sent to participants securely through a RedCap [19] platform to assign a daily vertigo severity score over a 24-day lead-in period prior to beginning the MBSR course. This lead-in period, where subjects were continued with conservative management alone, serves as an 'internal control', with which daily vertigo scores were compared after beginning the MBSR course. Subjects were asked to rate their level of vertigo over the prior 24-hour period (0 = none; 1 = mild; 2 = moderate; 3 = severe). Daily vertigo severity scores were then recorded throughout the eight-week course. Following completion of the course, the DHI, CFQ, PHQ9, GAD-7, VM-PATHI, and PROMIS surveys were completed.

Statistical analysis

Pre- and post-MBSR course dizziness and quality of life surveys were compared using Student's paired t-tests. Changes in pooled, mean daily vertigo severity scores were assessed using linear regression to determine if vertigo severity decreased after beginning the MBSR course. Effect sizes were calculated using Cohen's d-test. 

## Results

Twenty adult patients were enrolled in the trial (average age 46.7 years (range, 27-73 years); 20 (100%) females, 14 (70%) Caucasian). Subjects were, on average, highly educated (75% with bachelor's degree or higher), and a history of comorbid anxiety or depression was extremely common, occurring in 12 subjects (60%) and 10 subjects (50%), respectively (Table 1).

**Table 1:** Sociodemographic and clinical characteristics of study population (n=20) *Migraine prophylactic medications include tricyclic antidepressants, selective serotonin reuptake inhibitors, serotonin/norepinephrine reuptake inhibitors, etc. **Symptomatic treatment refers to medications with anti-nausea or anti-vertigo effects (benzodiazepine, 5-HT3 antagonists, antihistamines)

Characteristic	Prevalence; n (%) or avg (± SD)
Avg. age, years (± SD)	46.7 (± 16.3)
Sex	
Female	20 (100.0)
Male	0 (0.0)
Race	
Caucasian	14 (70.0)
Asian	4 (20.0)
Hispanic	2 (10.0)
Black	0 (0.0)
Education level	
Less than Bachelor's degree	5 (25.0)
Bachelor's degree	7 (35.0)
Graduate degree	8 (40.0)
Employment status	
Unemployed/retired	9 (45.0)
Full-time employment	11 (55.0)
Clinical Information	Prevalence (n (%))
History of depression	10 (50.0)
History of anxiety	12 (60.0)
History of anxiety and depression	8 (40.0)
Prior migraine prophylaxis*	4 (20.0)
Prior symptomatic treatment**	3 (15.0)

**Figure 2:**
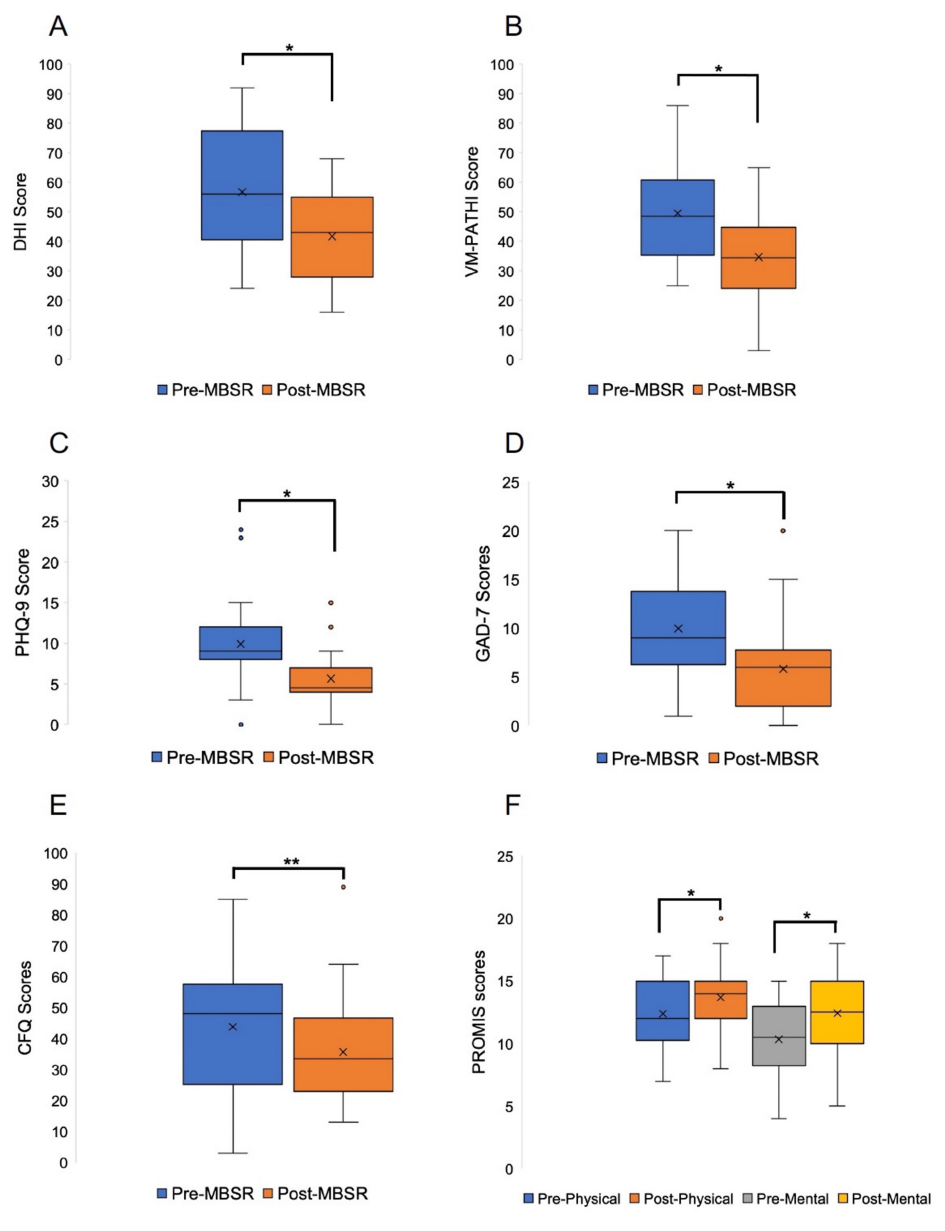
Improvement in dizziness symptoms and quality of life after participating in an eight-week mindfulness-based stress reduction (MBSR) course Box and whisker plots are shown comparing pre-MBSR and post-MBSR course treatment scores for A) Dizziness Handicap Inventory (DHI); B) the Vestibular Migraine Patient Assessment Tool and Handicap Inventory (VM-PATHI); C) Patient Health Questionnaire-9 (PHQ-9); D) General Anxiety Disorders-7 (GAD-7); E) Cognitive Failures Questionnaire (CFQ); and F) Patient Reported Outcome Measure Information System (PROMIS) Global Physical and Mental Health Forms. The mean is denoted by a black “x.” Note that lower scores for A through E represent improvement in symptoms while higher scores in F represent improvement in symptoms. Effect sizes: DHI = 0.98; VM-PATHI = 0.98; PHQ-9 = 1.21; GAD-7 = 0.82; CFQ = 0.44; PROMIS physical = 0.46; PROMIS mental = 0.7. * = p<0.01 by T-test. ** = p=0.02 by T-test.

Dizziness, as measured by DHI scores, decreased from an average of 56.7 to 41.7 after participation in the MBSR course (p<0.001, effect size = 0.98). Vestibular migraine-specific symptoms, as measured by the VM-PATHI questionnaire (18), showed a significant reduction after completion of the MBSR course (from an average of 49.5 to 34.7 (p<0.001, effect size = 0.98)). In addition, measures of general unhealthiness (PHQ-9) and cognitive effects of dizziness (CFQ) also reduced significantly after the MBSR course compared to pre-treatment, as did the level of pre-treatment anxiety, as measured by the GAD-7. Lastly, when measuring the global quality of life scores using PROMIS, mental and physical scores significantly improved from before the MBSR course to after (Figure 2).

**Figure 3:**
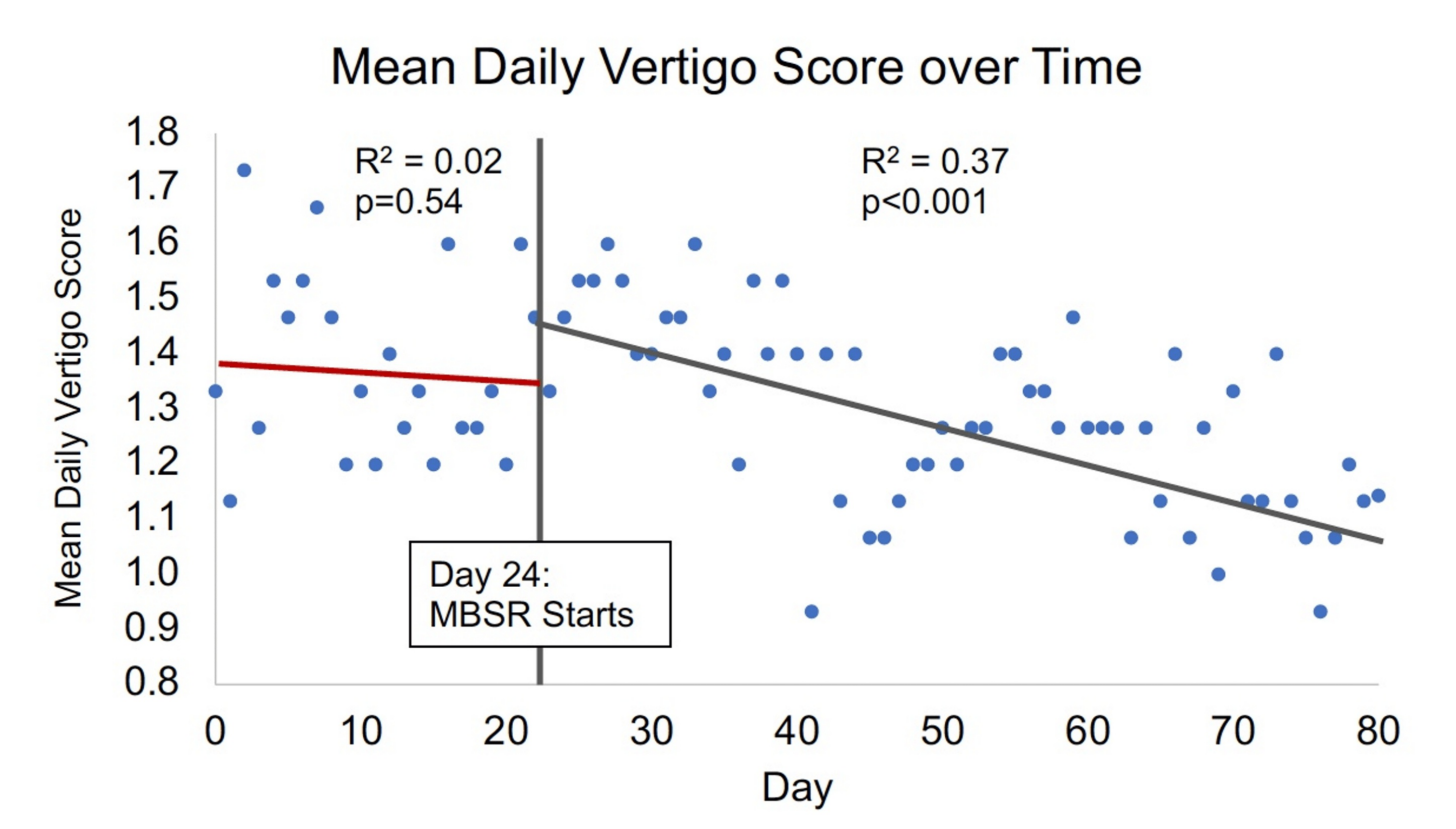
Correlation between mean daily vertigo scores and days of participation in the MBSR course 0=no vertigo, 1=mild, 2=moderate, 3=severe Note that daily vertigo scores were collected prior to the start of the MBSR course, which started on Day 24. MBSR - mindfulness-based stress reduction

Mean daily vertigo scores did not change significantly over time in the 24-day lead-in period (adjusted r2 = 0.03; p=0.54), indicating relative stability in dizziness symptoms over an approximately three-week period. However, mean daily vertigo severity scores decreased significantly over the eight-week MBSR treatment period (adjusted r2 = 0.32, p<0.001; Figure 3).

## Discussion

The traditional framework for treating VM includes identification and avoidance of dietary and lifestyle triggers for migraine as well as trials of prophylactic medications aimed at reducing the severity and frequency of migraines, including tricyclic antidepressants, mixed serotonin/norepinephrine reuptake inhibitors, antiepileptic medications, beta-blockers, and calcium channel blockers. Our work represents a novel departure from historical treatment strategies for VM by adding mindfulness-based stress reduction (MBSR) techniques to the standard of care for VM. We demonstrated that a guided, short-term MBSR course effectively decreased the burden of dizziness and vertigo symptoms in a cohort of subjects with VM. Through the completion of validated questionnaires, we additionally showed that measures of unhealthiness, depression, anxiety, and cognitive failures were all significantly reduced, with a concomitant increase in overall quality of life for those with VM.

DHI values decreased from an average of 56.7 pre-MBSR to 41.7 post-MBSR. Though both of these values indicate a moderately severe dizziness handicap (DHI>60 is the cutoff for "severe" dizziness), this represents both a statistically significant and clinically meaningful reduction in dizziness. Likewise, VM-PATHI scores decreased from 49.5 pre-MBSR to 34.7 post-MBSR. The magnitude of reduction of DHI and VM-PATHI scores in the present study was greater than in a recent trial in which 34 patients were treated with a mix of vestibular rehabilitation, pharmacoptherapy, and MBSR (average decrease in DHI and VM-PATHI of 57.4 to 47.2 and 49.7 to 39.0, respectively) [20]. DHI reduction in the current study (~15 points) was similar to DHI reduction in a recent study of vestibular rehabilitation alone for vestibular migraine (~18 points) [21].

Previously hypothesized pathophysiologic mechanisms for migraine and dizziness symptoms include spreading depression, hypersensitivity to cortical stimuli, and trigeminovascular inflammation via Substance P and CGRP activation [5,22]. In addition, vestibular migraineurs also seem to suffer from abnormal or heightened central processing of various sensory and pain stimuli that could serve to "prime" an individual for vestibular hypersensitivity. For example, the vestibular thalamus and cortex have been identified as components of the central vestibular network in a prior imaging study by Lopez et al. [23]. Abnormal thalamocortical activity is postulated to lead to hyperresponsiveness of sensory cortices and abnormal pain processing in patients with migraines [24]. Hypersensitivity of the ventral posterolateral and ventral posteromedial thalamic nuclei, associated with joint position send trigeminothalamic input, respectively, are hypothesized to be sensitized during a migraine attack and contribute to heightened sensory perception [25]. Finally, central nervous system hypersensitization to pain, associated with pain disorders, has also been found to occur during migraine attacks [24,26]. Our group and many others [14,15,27] have shown how commonly comorbid depression, anxiety, and stress exist in migraineurs and, in many cases, can exacerbate symptoms. Stress seems to be integral to the pathophysiology of migraine, as prior reviews of migraine have identified stress as the most common migraine trigger, with prevalence estimates of 36-72% reported [27]. Thus, at its core, the perception of vestibular symptoms related to VM is at least modulated by a global stress response that manifests as measurable changes in brain activity on imaging and electrophysiologic studies.

As a self-meditation technique, MBSR counters the limitations imposed by high-stress levels by teaching people to acquire three states of mindfulness: 1) intention, 2) attention, and 3) attitude. By adopting these mechanisms in response to a new stressor, trigger, or event, mindfulness is achieved through a process that is termed "re-perceiving", an adjustment of a patient's prior perspective. Additional complementary mechanisms are 1) self-regulation, 2) values clarification, 3) cognitive, emotional, and behavioral flexibility, and 4) exposure [28]. Individuals in MBSR courses are instructed to focus their attention on the experience or sensation of discomfort and the associated thoughts and emotions in a nonjudgmental manner, distinguishing these sensations from the emotional reaction of suffering and distress. By encouraging individuals to observe the experience nonjudgmentally, exposure to and observation of the experience of discomfort is postulated to desensitize the discomfort and reduce the emotional response [29]. T­­­­hus, equipping vestibular migraineurs with techniques to quickly recognize and positively adapt to new symptoms of dizziness, vertigo, brain fog, mental slowing, and other common symptoms of VM might aid in halting the repetitive and cyclical nature of dizziness catastrophization, and could account for the dramatic reduction in dizziness scores and improvement in quality of life measures that we observed. Our results agree with those reported by Naber et al., who used a combined MBSR/cognitive behavioral therapy/vestibular physical therapy to treat individuals with a heterogeneous group of dizziness disorders and found increased measures of quality of life and decreased burden of dizziness symptoms [13]. Intuitively, the group of probable or definite vestibular migraineurs in our study might be particularly amenable to sustained improvements after participating in an MBSR course, though longer-term follow-up data is not yet available. 

As healthcare costs and psychotropic polypharmacy continue to increase in the US in both adult and pediatric populations, it is reasonable to appeal to time-honored, non-pharmacologic treatment options for patients who suffer from disease processes that are heavily modulated or exacerbated by psychiatric comorbidities, such as depression and anxiety. In an environment where tertiary medicine continues to transition to multidisciplinary models for treatment, the addition of nonpharmacologic techniques such as MBSR could represent a critical piece to maximizing patient quality of life with this common and chronic disorder. Though mitigation of stress through nonpharmacologic methods should have utility in treating VM through a useful reframing of self-injurious perceptions of the severity of dizziness, comorbid anxiety and depression should, of course, also be aggressively treated simultaneously. 

There are several unique strengths of this current study. First, this is a novel application of MBSR to specifically address dizziness symptoms in vestibular migraineurs. Second, we used a previously validated VM-specific score - the VM-PATHI - to show improvement in VM-specific domains after participation in this MBSR program. Lastly, as the current study utilized a virtual platform for MBSR course delivery, the potential for easy scalability of this intervention is high. This is particularly noteworthy because numerous academic medical centers, including the one that served as this study's setting, offer MBSR programs in a virtual setting. 

Several major limitations apply to this study. First, the lack of a control group substantially limits the interpretation of the significant decreases in DHI and VM-PATHI scores and improvement in quality of life measures, as it is possible that vestibular migraineurs who presented to our tertiary clinic who were not enrolled in this study could have improved with dietary and lifestyle modifications alone. Though the study initially intended to enroll a control group of subjects with conservative treatment only, there was an unacceptably high rate of refusal to participate in the study when presented with a randomization strategy into MBSR versus no MBSR. Thus, given the desire to collect data in a reasonable amount of time, we instead followed a prospective, uncontrolled study design. Future studies will need to become multi-centered to accrue a sufficient number of control subjects. Secondly, this is a small study and included only 20 subjects, all of whom were female, middle-aged, highly educated, and of a high socioeconomic status. Thus, the generalizability of the results is limited to those with access to high-speed internet as well as a technological literacy that is required to participate in virtual sessions, in addition to the time required to participate in an intensive eight-week course. Others have argued that virtual or internet-based therapies risk being exclusionary to certain segments of the population without access to computers and high-speed internet or the basic skills required to navigate online courses [30]. However, this can be circumvented by simultaneously offering both in-person and remote courses, which is now the case at the study institution. Lastly, as this was a pilot study, we were unable to provide long-term follow-up data, so the question of whether or not these improvements are durable beyond eight weeks has not yet been answered. A multi-site, prospective, randomized trial with age-matched controls will be crucial to determine the effect size of MBSR for dizziness symptoms in VM. Additionally, there is a need for randomized controlled trials comparing MBSR to standard VM treatments (e.g., pharmacotherapy or vestibular rehabilitation) in order to establish the relative efficacy of MBSR. Long-term efficacy and sustainability remain to be determined through future studies.

## Conclusions

In this prospective, single-arm pilot study, a short-term mindfulness and meditation course was highly effective for decreasing dizziness burden and improving measures of quality of life in subjects with VM. Results from this study provide a platform on which future randomized, controlled trials can be conducted, to further characterize efficacy and sustainability of symptom reduction in VM.
